# Melanoma Patients with Unknown Primary Site or Nodal Recurrence after Initial Diagnosis Have a Favourable Survival Compared to Those with Synchronous Lymph Node Metastasis and Primary Tumour

**DOI:** 10.1371/journal.pone.0066953

**Published:** 2013-06-25

**Authors:** Benjamin Weide, Christine Faller, Margrit Elsässer, Petra Büttner, Annette Pflugfelder, Ulrike Leiter, Thomas Kurt Eigentler, Jürgen Bauer, Friedegund Meier, Claus Garbe

**Affiliations:** 1 Center for Dermatooncology, Department of Dermatology, University Medical Center, Tuebingen, Germany; 2 Skin Cancer Research Group, School of Public Health, Tropical Medicine and Rehabilitation Sciences, James Cook University, Townsville, Australia; University of Tennessee, United States of America

## Abstract

**Background:**

A direct comparison of prognosis between patients with regional lymph node metastases (LNM) detected synchronously with the primary melanoma (primary LNM), patients who developed their first LNM subsequently (secondary LNM) and those with initial LNM in melanoma with unknown primary site (MUP) is missing thus far.

**Patients and Methods:**

Survival of 498 patients was calculated from the time point of the first macroscopic LNM using Kaplan Meier and multivariate Cox hazard regression analysis.

**Results:**

Patients with secondary LNM (HR = 0.67; *p = *0.009) and those with initial LNM in MUP (HR = 0.45; *p = *0.008) had a better prognosis compared to patients with primary LNM (median survival time 52 and 65 vs. 24 months, respectively). A high number of involved nodes, the presence of in-transit/satellite metastases and male gender had an additional independent unfavourable effect.

**Conclusions:**

Survival of patients with LNM in MUP and with secondary LNM is similar and considerably more favourable compared to those with primary LNM. This difference needs to be considered during patient counselling and for stratification purposes in clinical trials. The assumption of an immune privilege of patients with MUP which is responsible for rejection of the primary melanoma, and results in a favourable prognosis is not supported by our data.

## Introduction

The prognosis of melanoma patients with loco-regional metastasis varies considerably with 5-year survival rates ranging between 29% and 51.6% [1]. The American Joint Committee on Cancer (AJCC) 2009 staging system includes the number of tumour-bearing nodes, the tumour burden at the time of lymph-node staging (microscopic vs. macroscopic), ulceration of the primary melanoma and the presence of in-transit or satellite metastasis to assign patients to the prognostic sub-stages IIIA-C [2]. The sub-stage is of major relevance with regards to patient selection for adjuvant therapies, planning of surveillance programs and for stratification purposes in clinical trials [3], [4]. Moreover, the assignation to a sub-stage is used for patient counselling and prognosis prediction [5], [6].

The AJCC stage III classification is exclusively based on the analysis of patients with cutaneous melanoma and lymph node metastasis (LNM) already present at the time of the initial melanoma diagnosis (referred to as primary LNM) [2], [7]. Nevertheless, the same stage III classification algorithm is also applied for stage I/II patients at the time point when loco-regional metastasis occur in the years after initial diagnosis (referred to as secondary LNM). Differences in prognosis were previously reported in patients with primary vs. secondary LNM but only small selected cohorts were analyzed [8], [9]. Moreover, differences of prognosis between patients with primary and secondary LNM might be assumed based on the observation that a long disease-free interval was found to be prognostically favourable for the subsequent course of disease in patients with recurrences [10]–[13].

The occurrence of metastases in the absence of an apparent primary tumour in 2% to 10% of melanoma cases is still an unexplained phenomenon.[14]–[16] One hypothesis is that an initially unrecognized primary melanoma regressed over time through immunological rejection [17] and is therefore not detectable at the time of melanoma diagnosis based on histopathology of a excised palpable node [18]. In several studies a more favourable prognosis was observed in MUP patients compared to those with known primary tumour [19]–[23]. A comparison between all three situations (primary LNM vs. secondary LNM vs. initial LNM in MUP) by Sondak *et al.* showed poorer survival of patients with primary LNM, as presented at annual meeting of ASCO 2010 but not yet published in detail [24].

The present study aimed to investigate prognostic factors of melanoma patients at the time of the first nodal macro-metastasis to identify potential differences between patients with metastases already present at initial melanoma diagnosis, those who metastasized subsequently during surveillance and patients with unknown primary melanoma.

## Methods

### Ethics Statement

All had given their written informed consent to have clinical data recorded by the Central Malignant Melanoma Registry (CMMR) registry. The institutional ethics committee Tübingen approved the study (identifier 144/2013R).

### Patients

Patients with cutaneous melanoma and nodal metastasis treated between 1996 and 2010 at the University Department of Dermatology in Tübingen, Germany, were identified in the Central Malignant Melanoma Registry (CMMR) database which prospectively records patients from more than 60 dermatological centers in Germany. The aims and methods of data collection by the CMMR have previously been reported in detail [3], [4], [25]. Of 792 patients with follow-up those initially presenting with micro-metastases only detected in sentinel node biopsy (294 patients) were excluded after individual file review resulting in a final sample size of 498.

Data obtained for each patient included gender, age at diagnosis of nodal disease, the situation (primary LNM vs. secondary LNM vs. initial LNM in MUP), presence of satellite/in-transit lesions, the number of involved nodes (1 vs. 2 or 3 vs. 4 or more) after the initial lymph node staging procedures, the date of the initial diagnosis and the last follow-up, and the date and cause of death, if applicable. The following characteristics of the primary tumour were analyzed: body site (axial vs. extremities), Breslows tumour thickness, Clarks level of invasion (I-III vs. IV, V), ulceration, and subtype (superficial spreading melanoma vs. nodular melanoma vs. lentigo maligna melanoma vs. acral lentiginous melanoma).

### Statistical Analysis

Follow-up time was defined from the date of diagnosis of the first lymph node metastasis to the date of last follow-up or death. Estimates of cumulative survival probabilities according to Kaplan-Meier were described together with 95% confidence intervals and compared using two-sided log-rank test statistics. Median survival times (MST) are presented. For the analysis of disease-specific survival patients who were alive at the last follow-up or died without evidence of metastatic melanoma were censored.

Multivariable Cox proportional hazard models were used to determine independent prognostic factors. Categorized variables were dummy coded to adhere to the linearity assumption of multivariable regression analysis. All characteristics described above were considered in multivariable analysis. Missing values were assessed independently as a separate group to allow the assessment of patients with MUP. Forward and backward stepwise procedures of the multivariable modelling process were conducted. Results of the Cox models were described by means of hazard ratios (HR) together with 95% confidence intervals (95% CI), p-values were based on the Wald test. Confounding was assessed by checking the effect of each remaining non-significant variable, which was not in a model, on factors in the model. If changes in the estimate of factors in the model of 5% or more occurred the variable was considered a confounder. Differences in the distribution of variables according to the situation at first occurrence of metastasis were calculated by Fisheŕs exact test. Throughout the analysis, p values less than 0.05 were considered as statistically significant. All statistical analyses were carried out using the SPSS Version 19 (IBM SPSS, Chicago, Illinois, USA).

## Results

### Patients

Patientś characteristics are shown in Table 1. A total of 498 melanoma patients (58% male) were included in the survival analysis at the time of initial stage III diagnosis. The median age was 59 years (inter quartile range [IQR] 47–70 years). The median follow-up time for patients who died was 20 months (IQR 11–35) and 58 months (IQR: 25–104) for patients who were alive at the last date of observation. The median survival time according to Kaplan Meier (MST) was 42 months. Cumulative survival rates were 65.0% (2 years), 44.4% (5 years) and 36.1% (10 years).

**Table 1:** Patient characteristics of 498 patients with lymph node metastases.

Characteristic	Entire cohort (n = 498)		Primary LNM(n = 111)	Secondary LNM (n = 319)	LNM in MUP (n = 68)	
	N	%	%	%	%	p#
Age at stage III diagnosis						0.136
<50 years	150	30.1	23.4	31.0	36.8	
50–59 years	100	20.1	16.2	20.4	25.0	
60–69 years	119	23.9	26.1	24.5	17.6	
≥70 years	129	25.9	34.2	24.1	20.6	
Gender						0.024
Male	291	58.4	67.6	53.9	64.7	
Female	207	41.6	32.4	46.1	35.3	
Tumour thickness						<0.001
<1.00 mm	83	20.3	7.6	29.3		
1.00–1.99 mm	102	24.9	6.7	29.3		
2.00–3.99 mm	114	27.9	33.3	27.3		
≥4.00 mm	110	26.9	52.4	14.1		
na	89					
Ulceration						<0.001
Yes	153	39.3	38.5	68.8		
No	236	60.7	61.5	31.2		
na	109					
N stage						0.059
N1b	238	49.3	38.0	53.7	46.9	
N2b	113	23.4	25.9	21.9	26.6	
N3	132	27.3	36.1	24.4	26.6	
na	15					
Number of involved lymph nodes						0.365
1	265	55.1	48.6	58.1	51.6	
2–3	131	27.2	31.8	24.5	32.8	
4 or more	85	17.7	19.6	17.4	15.6	
na	17					
Sub-stage						<0.001
IIIB	165	40.3	24.0	48.6	na*	
IIIC	244	59.7	76.0	51.4	na*	
na	89					
Metastases						
LNM only	438	55.4	79.3	91.5	85.3	0.003
LNM plus satellite/In-transit metastases	60	7.6	20.7	8.5	14.7	

# p-value indicating differences in distribution according to the situation at first occurrence of metastasis (missing values are not considered).

* not classified due to missing ulceration data. na = missing values; LNM = lymph node metastasis; MUP = melanoma of unknown primary.

64.1% of patients developed the first nodal metastasis after the excision of the primary melanoma in contrast to 22.2% who already presented with metastases at the initial diagnosis and 13.7% had an unknown primary tumour. Significant differences were observed between these subpopulations regarding gender, tumour thickness and ulceration of the primary melanoma, sub-stage and the presence of satellite/in-transit metastasis (Table 1).

### Survival Analysis of Patients with Lymph Node Metastases

**Figure 1:**
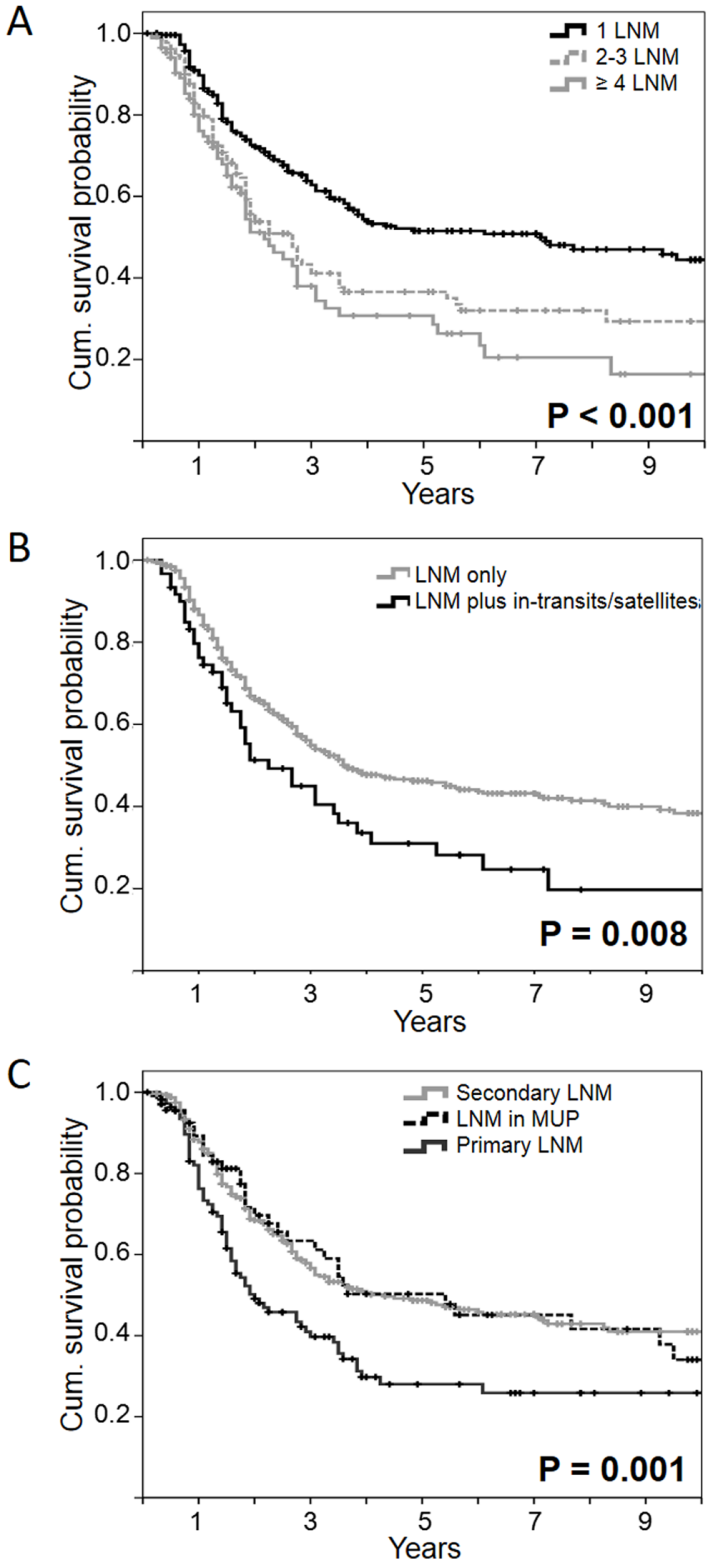
Survival according to Kaplan Meier. Differences were observed according to the number of lymph node metastases (LNM) (A) and presence or absence of in-transit or satellite metastases (B). Survival is more favourable for patients with secondary LNM or lymph node metastases but unknown primary tumour (MUP) compared to patients with primary LNM (C).

In bivariate analysis, shorter survival was observed in patients with more than one involved lymph node (Figure 1A), if in-transit/satellite metastases were present (Figure 1B) and in male patients. Multivariate analysis showed that the impact was strongest in patients with more than 3 lymph node metastases (HR = 2.2; *p<*0.001) or 2–3 lymph node metastases (HR = 1.6; *p = *0.002) followed by the presence of in-transit metastases (HR = 1.6; *p = *0.012) and male gender (HR = 1.4; *p = *0.023). Interestingly, patients with secondary LNM and MUP patients had a considerably better prognosis compared to initially metastasized patients with known primary tumour both in bivariate (Table 2) and multivariable analysis (Table 3). The median survival time (MST) was 24 months for patients with primary LNM, 52 months for those with secondary LNM and 65 months for MUP, respectively (Figure 1C). Age, body site and histopathological characteristics of the primary melanoma did not influence survival according to our analysis (Table 2).

**Table 2:** Survival analysis of 498 patients with palpable lymph node metastases based on Kaplan Meier.

Factor	n	%	% Dead	5 Year survival rate[95%-CI*] (%)		10 Year survival rate[95%-CI*] (%)		p**
Gender								0.012
Male	291	58.4	55	38.9	[32.6; 45.2]	31.4	[24.5; 38.3]	
Female	207	41.6	45.4	52.0	[44.4; 59.6]	42.8	[34.0; 51.6]	
Age								0.382
<60 years	250	50.2	54.4	42.2	[35.3; 49.1]	33.7	[26.4; 41.0]	
> = 60 years	248	49.8	47.6	46.8	[39.7; 53.9]	38.9	[30.7; 47.1]	
Body site of primary								0.562
Axial	226	45.4	52.2	43.5	[36.4; 50.6]	36.4	[28.4; 44.4]	
Extremities	204	41.0	51	43.3	[35.5; 51.1]	37.3	[29.3; 45.3]	
Missing data/unknown primary	68	13.7	47.1	50.2	[36.5; 63.9]	34.0	[18.7; 49.3]	
Ulceration of the primary								0.054
Not ulcerated	236	47.4	48.7	47.4	[40.3; 54.5]	42.2	[34.6; 49.8]	
Ulcerated	153	30.7	53.6	38.3	[30.7; 45.9]	31.2	[21.2; 41.2]	
Missing data/unknown primary	109	21.9	52.3	46.0	[35.4; 56.6]	31.1	[19.7; 42.5]	
Histologic subtype of primary								0.228
SSM	197	39.6	48.7	47.5	[39.9; 55.1]	42.4	[34.4; 50.4]	
Nodular	119	23.9	58.0	40.6	[32.6; 48.6]	30.6	[19.6; 41.6]	
LMM	19	3.8	47.4	53.6	[29.3; 77.9]	42.9	[15.9; 69.9]	
ALM	45	9.0	53.3	21.9	[3.5; 40.3]	0.0	[0.0; 0.0]	
Missing data/unknown primary	118	23.7	47.5	46.8	[36.4; 57.2]	35.3	[23.1; 47.5]	
Clark’s level of invasion								0.364
Level I–III	99	19.9	50.5	45.5	[34.9; 56.1]	41.6	[30.6; 52.6]	
Level IV–V	243	48.8	52.3	43.6	[32.6; 54.6]	35.3	[27.5; 43.1]	
Data Missing/unknown primary	156	31.3	49.4	44.9	[35.9; 53.9]	33.5	[23.3; 43.7]	
Breslow’s thickness of primary								0.186
<2 mm	185	37.1	49.7	47.0	[39.2; 54.8]	40.6	[32.0; 49.2]	
> = 2 mm	224	45.0	53.1	40.2	[31.6; 48.8]	33.9	[26.1; 41.7]	
Missing data/unknown primary	89	17.9	48.3	48.7	[36.7; 60.7]	34.5	[21.4; 47.6]	
Situation at first occurrence of metastasis								0.001
Primary LNM	111	22.2	62.2	28.0	[18.2; 37.8]	25.8	[16.0; 35.6]	
Secondary LNM	319	64.1	48.0	48.7	[38.9; 58.5]	41.0	[34.3; 47.7]	
Initial LNM but MUP	68	13.7	47.1	50.2	[36.5; 63.9]	34.0	[18.7; 49.3]	
Satellite or Intransit metastasis								0.008
Present	60	12.0	65.0	31.0	[17.9; 44.1]	19.7	[6.2; 33.2]	
Absent	438	88.0	49.1	46.2	[32.7; 59.7]	38.3	[32.4; 44.2]	
Number of involved lymph nodes								<0.001
1	265	53.2	43.8	51.5	[44.8; 58.2]	44.5	[36.9; 52.1]	
2–3	131	26.3	58.0	36.6	[29.0; 44.2]	29.3	[19.3; 39.3]	
4 ore more	85	17.1	62.4	30.7	[19.3; 42.1]	16.4	[4.8; 28.0]	
Missing data	17	3.4	52.9	54.8	[29.7; 79.9]	45.6	[19.1; 72.1]	

* 95%-CI = 95% confidence interval;

** p-values are results of log rank tests excluding cases with missing values.

LNM = lymph node metastasis; MUP = melanoma of unknown primary.

**Table 3:** Independent prognostic factors for 497 patients with macroscopic lymph node metastases according to the multivariable Cox proportional hazard analysis.

Prognostic factor	Sample size (n = 497*)	% Dead**	Relative risk (95% CI)#	p-value
Gender				
Female	207 (41.6%)	44.4%	1	
Male	290 (58.4%)	54.1%	1.4 (1.04, 1.8)	*p = *0.023
Situation at first occurrence of metastasis				
Primary LNM	110 (22.1%)	61.8%	1	
Secondary LNM	319 (64.2%)	47.0%	0.67 (0.49, 0.91)	*p = *0.009
Initial LNM in MUP	68 (13.7%)	45.6%	0.45 (0.25, 0.82)	*p = *0.008
In-transit metastases				
No	437 (87.9%)	48.5%	1	
Yes	60 (12.1%)	61.7%	1.6 (1.1, 2.2)	*p = *0.012
Number of positive lymph nodes				
One	281 (56.5%)	43.8%	1	
Two or three	131 (26.4%)	56.5%	1.6 (1.2, 2.2)	*p = *0.002
Four or more	85 (17.1%)	61.2%	2.2 (1.6, 3.0)	*p<*0.001

# 95% CI = 95% confidence interval;

* One patient was censored before any death occurred and was automatically removed from the analysis;

** Disease-specific death; the model was adjusted for the confounding effects of ulceration of primary tumour, age of the patient at diagnosis and for missing values for the number of positive lymph nodes (n = 17).

LNM = lymph node metastasis; MUP = melanoma of unknown primary.

## Discussion

We confirmed the important prognostic role of the number of involved nodes and the presence of in-transit/satellite metastases in our study of melanoma patients with macroscopic lymph node metastases. In contrast to others, we also included stage I/II patients who relapsed to the lymph nodes in the years after the initial melanoma diagnosis and patients with unknown primary melanoma in our study. Only 51% of all 792 stage III patients with nodal disease treated at our institution between 1996 and 2011 presented with LNM at the time of initial diagnosis, while 40% represent relapsed stage I/II patients and 9% were patients with melanoma of unknown primary.

We found that patients with secondary LNM had a better prognosis (HR 0.67; p = 0.009) compared to patients with LNM detected at the time of primary melanoma excision as described before [8], [9]. Multivariate analysis, which was performed to investigate the relative impact of different prognostic factors and their interaction by confounding showed that the impact on prognosis was independent from all other analysed factors. This was of major importance as differences in the distribution of other variables were detected between patients with primary LNM, secondary LNM and MUP (Table 1). The observation that a long disease-free interval before recurrence is associated with a favourable subsequent course of disease provides further indirect evidence that the timing of lymph node involvement might be relevant for prognosis [10]–[13], [25], [26]. Disparate results about the influence of this time interval were only reported in a minority of studies [27]–[29]. There are different possibilities to explain a favourable survival of patients with secondary vs. primary LNM: (a) the disease-free interval might reflect differences in the biologic behaviour and aggressiveness of tumour cells suggesting a favourable prognosis of patients with secondary LNM [30]. Patients with primary LNM might have a more aggressive type of tumour, which is capable of metastasizing early. This is supported by the observations of a higher tumour thickness and a higher frequency of additional satellite/in-transit metastasis in patients with primary LNM. These patients might also have a worse prognosis in the subsequent course of the disease. In contrast, having “silenced” tumour cells growing to clinically detectable metastases only after several years might be prognostically favoured even after loco-regional metastasis has occurred. Ulceration of the primary melanoma was mainly observed in patients with secondary LNM, but only present in a subpopulation of patients in LNM already detected at the time-point of initial diagnosis (68.8% vs. 38.5%; p<0.001). Even if ulceration had not prognostic impact in the entire patient cohort its significant correlation with secondary lymph node metastasis was unexpected and indicates its biologic relevance, which needs to be further investigated in future studies.

(b) The favourable prognosis of relapsed stage I/II patients might reflect the efficiency of surveillance programs aiming at early detection of recurrences in the years after excision of the primary melanoma. Recent studies found a survival benefit for patients under surveillance beyond lead time bias [3]. Nevertheless, the differences in survival between patients with primary and secondary LNM or MUP could also be influenced by other factors not considered in our study. Tumour growths dynamics represented by the tumour doubling time [31] and/or mitotic activity and the host immune response e.g. the intensity of the lymphocytic infiltrate in melanoma tissue [32] might be relevant for prognosis as well and could be differentially distributed between the subpopulations. An analysis of these histopathological features was not performed because tissue blocks were not available.

Our patients with initial lymph node metastasis but unknown primary melanoma had a better prognosis than patients with primary LNM and known primary tumour (HR = 0.45; p = 0.008). This is in contrast to a few prior reports [33]–[35] but in agreement with most other publications in this field [19]–[22]. Moreover, prognosis of MUP patients resembles that of patients with secondary LNM (Figure 1C). There is only one study by Sondak *et al.* comparing primary LNM, secondary LNM and initial lymph node metastasis in MUP in a comprehensive analysis, implying very similar findings, but thus far detailed data have not been published [24].

The comparison of these three clinical situations regarding prognosis might help to understand the phenomena of MUP. A regression zone is frequently observed in primary tumours and complete regressions of primary melanomas as postulated in MUP could occasionally be demonstrated [36], [37]. The reasons for regression of the primary tumour are incompletely understood but an effective immune response directed against melanoma cells was suggested as an explanation for local regression [17]. Whether or not this immune response might be systemically relevant and beneficial for the further course of disease in these patients is unclear, because underestimation of the tumour thickness due to regression hampers the investigation of its prognostic impact [37]. Lee *et al* [19] and Prens *et al* [21] both demonstrated a clear survival advantage of patients with MUP after therapeutic lymphadenectomy compared to patients with known primary tumour and explained this observation with a strong endogenous immune response directed against melanoma resulting in both regression of the primary tumour and a better outcome. On the other hand, Shaw *et al.* suggested that the immune response leading to regression does not reflect a privilege but is a secondary activation of the immune system indicating metastatic spread of melanoma cells to the lymph nodes [38].

We agree that MUP might be regarded as a combination of a regressed primary melanoma with the subsequent occurrence of secondary lymph node metastasis. But on the other hand, patients with MUP and those with secondary LNM but known primary tumour had a similar survival in our study. The comparison of prognosis between these two situations according to our analysis is not suggestive for a clinically relevant immune advantage in MUP and in agreement with prior studies [16], [39].

Ulceration had no impact on prognosis in our patients. This is in agreement with the stage III analysis of Balch *et al.* finding ulceration and other histopathological characteristics to be less important prognostic markers in patients with macroscopic nodal disease compared to those with micro-metastases only [1]. A favourable prognosis of melanoma patients at the time of the first nodal metastasis was associated with female gender in bivariate and multivariable analyses. Interestingly, this association was limited to patients with secondary LNM and MUP. It was not observed in our patients with primary palpable LNM as described by others [1]. In general, there are conflicting results regarding the prognostic role of gender in loco-regionally metastasized patients in the literature [27], [40]–[43]. Our results are similar to those reported by Nowecki *et al*., who analyzed prognostic factors in 286 melanoma patients after therapeutic lymphadenectomy and found that a high number of involved nodes, an extracapsular lymph node involvement and male gender were independently associated with poor survival. They observed a better survival in patients with metastasis occurring later than 24 months after excision of the primary melanoma compared to those with metastases appearing earlier, but did not include patients with MUP [12].

### Conclusion

A high number of involved nodes, the presence of additional in-transit/satellite metastases and male gender were negative predictors of survival after occurrence of macroscopic lymph node metastasis including patients with unknown primary tumour and those with secondary LNM. In contrast to patients with known primary melanoma who already presented with lymph node metastases at initial diagnosis, prognosis in both other groups was more favourable (MST 65 months for MUP and 52 months for secondary LNM vs. 24 months for primary LNM). This difference needs to be considered for patient counselling and for stratification purposes in clinical trials. The assumption of an immune advantage in patients with MUP which is responsible for rejection of the primary melanoma and results in a more favourable prognosis is not supported by our data, as prognosis of patients with MUP is similar to that of stage I/II patients with recurrent nodal disease.
